# Saving an Old Tooth

**Published:** 1898-03

**Authors:** Ernest M'Caffey


ARTICLE XII.

### SAVING AN OLD TOOTH.

BY ERNEST M'CAFFEY.

The doctor stretched my mouth open until he could see my heart beat. Then he said, cautiously, "I think we can save that tooth." Running a long, inquisitive wire into the tooth to the depth of about sixteen inches, he stirred up something known as a "nerve." In response to my fervent yell he asked, "Does that hurt?" I wiped tears out of my eyes and simply said "Yes." Then he got some zahnarzt crowbars and pried here and there at the tooth, occasionally waking up that cussed nerve, which had been growling ever since at its unceremonious handling. Then he said: "We will have to kill the nerve first, and fill the tooth afterwards."

"How long will that take?" I asked anxiously.

"On, you can't tell about that," he replied; you can have it pulled if you prefer it." he added. I went to the glass and smiled at myself. It was as I feared. If that tooth was yanked out, my smile was ruined.

There is nothing so unpleasant as a foreshortened smile. The absence of the tooth at either end gives it a wolfish character. A tooth out of the center of a smile simply wrecks it. No, I could not afford to have that tooth pulled.

Stimulated then by the strongest ingredient in the mal-character—viz., vanity—I resolved to take my medicine, and so the pact was made.

I began to make regular trips to his office, and soon became inured to scenes that at first chilled the glad blood in my veins and caused the soft and insignificant goose pimples to start from my flesh as a trumpet call. People with a strained expression of countenance met there, and the doctor's little room, which I had mentally named the chamber of horrors, re-echoed with groans, yells, and imprecations. And yet he was a gentle-hearted man. He did not revel in those pandemoniums of whoops and sobs, but the stern necessities of dentistry compelled them.

His instruments of torture, called by courtesy dental tools, were many and varied. He was very skillful in his profession, and when he took a job, he did it in first class style. The dental tools are simply copies in miniature of articles used in the Spanish Inquisition and on refractory prisoners in the Tower of London. There are monkey-wrenches, raspers, files, gouges, cleavers, picks, squeezers, drills, daggers, little crowbars, punchers, chisels, pinchers, and long wire feelers with prehensile, palpitating tips that can reach down through the roots of a throbbing tooth and fish up a yell from your inner consciousness. When a painstaking dentist cannot hurt you with the cold steel, he lights a small alcohol lamp, and heats one of his little spades red hot, and hovers over you with an expectant smile. Then he deftly inserts this into your mouth, and when you give a yell, he says, "Does that hurt ?"

Well, the first thing to do was to kill the nerve. The nerve is a long, starved angle-worm growth that starts in the tooth somewhere and goes down and up with three distinct tentacles or feelers. One of these connects with your brain, one with your heart, and the other with your soul. Every time a nerve is touched an electric shock goes to each of these terminal points, and you feel as if you had been shot, stabbed, and burned with a hot iron at one and the same time.

The doctor toyed with this nerve of mine for some weeks. Whenever the combination was made I used to kick out with one foot and cry "Ow !" or grab his hand and say, appealingly, "Oh, don't!" Then he would say, "We won't hurt you."

Sometimes he would lull me into a fancied security, and I would be counting three hundred, or saying to myself, "Even this will pass away," when all of a sudden four thousand rattle snakes would dart their venom into me simultaneously, a hundred mules would kick me, a score of bumblebees would sock their stingers into me, and the world would come to an end. Then I would know that he had stepped on the nerve with the "teaser." The "teaser" is the boss "feeler," being fine as a horsehair and of the most undoubted yell-producing power.

After a long while the nerve capitulated. I had lost eleven pounds in the process, but a great gain had been made. The tooth was now as tender as a mush-and-milk poultice, and even to tickle it with an ostrich tip would produce exquisite agony. He used to soothe it from time to time with various lotions, and finally he began to quarry out the dead bone a little. This was gruesome work, for there were tender places all over the tooth, as thick as spots on a leopard, and every time he jammed a chisel into one of them I almost fainted. I was kept in a continuous cold sweat for weeks, thinking about it before I went, going through with it while I was at the office, and thinking about it after I had left.

After he had amused himself by digging and blasting out a lot of little galleries in the upper part of the tooth, he began to "treat" the root apertures.

This is a most ingenious and refined cruelty, and by some dentists is preferred even to nerve-killing. The process is to first feel around with the "teaser" on the sensitive roots; next to put a little cotton dipped in carbolic or nitric acid, creosote or turpentine around the "teaser," and stick it away down into the same place. This hurts powerfully. It cleanses the roots of impurities, though. A lighted match would be less painful, but the aperture is not large enough to admit one. After this some cotton filling is stuffed in, and you are told to come back in three days. That night you awake at twelve o'clock with "your soul in arms and eager for the fray." You dig out all the filling and pace up and down the room, saying at intervals, "Oh, gosh! why didn't I have it pulled?"

Then you go back and the treatment is renewed. The doctor varied the upper filling by putting in hard rubber filling after a while. This is put in soft and hot, and hardens when it gets cold.

When you wake up with this hurting you at night, you can't get it out. A red-hot hairpin may get some of it out, but you are liable to glance off and get your gums into the sear and yellow. So you generally recoup by taking the Lord's name, as some say, in vain, but it soothes you, anyway.

When the roots are ready to fill, a gladsome joy pervades your entire system. The birds sing, the skies are bright, roses bloom, men and women are better, the whole world has changed in the twinkling of an eye. The day the roots are filled you go home and kiss your mother, and eat your supper on both sides of your mouth. For, mark you, when a tooth is being filled, the jaw it adorns is practically sidetracked until the crucifixion is over.

The last scene in the drama is when the doctor put in the top filling. The roots had already been plugged with a red putty which had hardened into a regular tooth cement. I lay back in the chair and the tap, tap of the hammer and punch sounded as melodious as Joaquin Miller's line of "A woodpecker pounded a pine top shell."

I was wrapped in a dream of delicious joy. Not like some of my acquaintances would I be forced to launch myself into society with a fragmentary misfit smile. No, indeed! And when the whole thing was through, I shook the dock's hand, and he told me he had never meant all along to so kill me by inches, but that dentistry was dentistry.

Then I went to the glass and smiled.—*Chicago Herald.*

